# Machine Learning-Driven Prediction of Manufacturing Parameters and Analysis of Mechanical Properties of PC-ABS Specimens Produced by the Fused Deposition Modeling Additive Manufacturing Method

**DOI:** 10.3390/polym18070886

**Published:** 2026-04-04

**Authors:** Arda Pazarcıkcı, Koray Özsoy, Bekir Aksoy

**Affiliations:** 1 Department of Mechatronics Engineering, Faculty of Technology, Isparta University of Applied Sciences, 32100 Isparta, Türkiye; yl2330654007@isparta.edu.tr (A.P.); bekiraksoy@isparta.edu.tr (B.A.); 2 Department of Machine and Metal Technologies, Isparta OIZ Vocational School, Isparta University of Applied Sciences, 32230 Isparta, Türkiye

**Keywords:** PC-ABS, fused deposition modeling, additive manufacturing, tensile strength, flexural strength, impact energy, ANOVA, machine learning

## Abstract

This study aims to investigate the effect of manufacturing parameters on the mechanical properties of PC-ABS samples produced by the Fused Deposition Modeling (FDM) additive manufacturing method and to model these relationships using machine learning methods. In the study, the parameters of printing speed, infill density, and raster angle were determined according to the Taguchi L16 experimental design, and tensile, bending, and impact tests were performed on the produced samples. Experimental results showed that the infill density parameter resulted in an increase in tensile strength of approximately 62% (from 25.10 MPa to 40.71 MPa) and an increase in flexural strength of approximately 46% (from 45.13 MPa to 66.13 MPa). Furthermore, an improvement in impact energy of approximately 45% (from 1.698 J to 2.467 J) was achieved under optimum printing speed conditions. Mechanistic properties were predicted using Decision Tree, Random Forest, K-Nearest Neighbors, and Multilayer Perceptron models with a dataset generated from experimental data. Comparing the model performances, the Random Forest algorithm was found to provide the highest prediction performance with accuracy in the R^2^ range of 0.94–0.99 and RMSE values below 0.5, demonstrating strong generalization capabilities. The results showed that infill density is the most decisive parameter on both tensile and flexural strength, and that printing speed has a significant effect, especially on impact energy. ANOVA analyses revealed that all main parameters have statistically significant effects on mechanical properties. In the performance comparison of the machine learning models, the Random Forest algorithm provided the highest prediction accuracy, demonstrating that mechanical properties can be reliably predicted. In conclusion, it has been shown that the mechanical performance of PC-ABS parts produced by the FDM method can be optimized by using the correct selection of production parameters and machine learning-based modeling approaches.

## 1. Introduction

Additive manufacturing (AM) is an innovative technique that makes it possible to produce complex, durable, and lightweight parts by combining materials in layers [1,2]. In sectors such as aerospace, defense, and biomedicine, its ability to directly manufacture components makes this method advantageous compared to traditional manufacturing processes [3,4]. In line with these technological advancements, various additive manufacturing methods have been developed over time to cater to different material types, production needs, and precision levels [5]. Additive manufacturing technologies are classified according to the form of the material used and the layer deposition method; they are further subdivided into subclasses such as powder bed fusion [Selective Laser Melting (SLM), Electron Beam Melting (EBM), Direct Metal Laser Sintering (DMLS)] [6], material jetting, material extrusion [Fused Deposition Modeling (FDM)] [7], binder jetting, vat photopolymerization (SLA, DLP), sheet lamination (LOM), and directed energy deposition (DED), and are widely used in a variety of industrial fields [8,9]. Among these groups, the material extrusion technique, which is one of the most common and accessible methods, stands out especially with the Fused Deposition Modeling (FDM) method.

FDM, which works by depositing thermoplastic filaments layer by layer, is one of the most widely used additive manufacturing methods due to its advantages such as low cost, device portability, and lack of solvent requirement. The main reason for choosing this method stems from its high production speed, compatibility with a variety of materials, and rapid prototyping capabilities [10,11,12]. In the FDM method, polymer materials in fused filament form are widely used; filaments such as ABS, PLA, PETG, PA, PC, PE, PVA, TPU, ASA, and PEEK are prominent [13,14]. Among these materials, ABS is a versatile polymer preferred in engineering applications requiring high strength, thanks to its superior properties such as impact resistance, toughness, and machinability [15]. Productions carried out using the FDM method are widely used in various application areas such as the production of complex and lightweight parts in the aerospace sector, personalized implant design in the healthcare field, and prototyping, custom part and lightweight structure manufacturing in the automotive industry [16,17]. The flexibility and customizability offered by FDM technology play a significant role in this widespread use [18,19]. FDM technology, in addition to the advantages of rapid prototyping, also enables the production of high-performance and multifunctional materials with customizable geometries. Recent studies show that approaches such as hierarchical structuring and nanoparticle alignment in 3D printing processes can significantly improve functional properties such as electromagnetic shielding and thermal conductivity [20].

In this context, each of the parameters such as layer thickness, infill density, printing speed, table temperature, raster angle, part orientation, and nozzle diameter plays a decisive role in the quality, strength, and dimensional accuracy of the produced part [21,22]. Optimizing process parameters correctly, both technologically and economically, to produce parts with the desired mechanical properties increases production efficiency and ensures that parts meet functional and structural requirements [23,24]. The performance of parts produced by the FDM method is directly related not only to the process parameters but also to the physical and chemical properties of the material used. Accordingly, the use of artificial intelligence-based data analysis and prediction methods is becoming increasingly important to understand the effects of parameters on mechanical strength and to determine the most suitable combinations [25].

The integration of AI-based methods and machine learning (ML) technologies into manufacturing processes, particularly artificial neural networks (ANNs), Genetic Algorithms (GAs), and hybrid approaches, stands out as an effective optimization tool in modeling the complex and nonlinear relationships between numerous parameters in the filament extrusion process, where traditional methods are insufficient [17,26]. ML is an effective tool against the challenges in predicting mechanical performance due to the various materials and numerous parameters used in the FDM process, thanks to its ability to discover complex patterns [27]. ML is a subfield of artificial intelligence concerned with algorithm design and analysis, developing models that generate predictions and decisions by learning from data [28]. Advanced machine learning algorithms such as Linear Regression [29], Ridge Regression [30], K-Nearest Neighbor Regression [31], Random Forest Regression [32], Support Vector Regression [33], Gradient Boosting Regression [34], XGBoost [35], and Multilayer Perceptron Regression [36] are frequently used to achieve high accuracy in nonlinear data and offer effective solutions, especially in the modeling and optimization of additive manufacturing and complex production processes.

Machine learning algorithms are frequently used to predict the mechanical test performance of ABS specimens produced by FDM. Ramiah and Pandian analyzed experimental data obtained from tensile, bending, and impact tests using a Decision Tree algorithm, identifying key patterns in material performance. Processing parameters such as raster angle, raster width, and layer thickness were optimized to maximize mechanical properties. It was concluded that the appropriate selection of these parameters facilitated prototype production [37]. Özkül et al. used twenty-five different machine learning algorithms to predict the mechanical properties of ABS samples produced by FDM, optimizing layer thickness, infill density, and nozzle temperature. The results showed that material savings and predictable mechanical performance can be achieved with the right algorithm and data processing [36]. Veeman et al. compared machine learning algorithms, including Linear Regression, Decision Trees, Random Forests, and AdaBoost, to predict the Rockwell hardness of ABS samples produced by FDM, with Random Forest yielding the best performance (R^2^ = 0.986) [38]. The study showed that mechanical properties can be predicted with high accuracy by correctly adjusting the printing parameters and selecting the appropriate model. Panico et al. varied process parameters such as infill pattern, extrusion temperature, printing speed, and layer thickness in ABS samples produced by the FDM method [24]. The relationships of these parameters with tensile strength, elastic modulus, and elongation under maximum stress were analyzed using the Random Forest algorithm. As a result of the analyses, R^2^ values of 93% for tensile strength, 87% for elastic modulus, and 82% for elongation under maximum stress were obtained. These results show that appropriate selection of process parameters provides significant improvements in production process optimization. Kumar et al. used a Decision Tree algorithm to estimate the tensile stress of ABS samples produced by FDM, with the model achieving an accuracy of R^2^ = 0.74 on the test data. The highest stress was obtained with 39 MPa at 60% filler and a nozzle temperature of 250 °C [39]. The study shows that the appropriate selection of process parameters, especially filler density, is decisive in determining the mechanical properties. Table 1 summarizes the manufacturing parameters and mechanical property data of FDM-produced PC-ABS specimens used for machine learning analysis.

The main objective of this study is to model the relationships between manufacturing parameters and mechanical test results in PC-ABS samples produced by the FDM method using machine learning algorithms, thus making the production process more predictable and optimized. In this context, printing speed, infill density, and raster angle were determined using the Taguchi L16 experimental design to investigate the effect of manufacturing parameters on PC-ABS filament. Infill density directly controls the internal structure and load-bearing capacity, while printing speed affects interlayer bonding and energy absorption. Scanning angle determines the anisotropic mechanical behavior of the printed components. Therefore, these parameters were chosen because they are among the most important factors affecting the mechanical performance of parts produced with FDM. The produced samples were then subjected to tensile, bending, and impact tests. A dataset was created using the data obtained from these tests. Mechanical strengths were predicted using Decision Tree, Random Forest, K-Nearest Neighbors, and Multilayer Perceptron algorithms on this dataset, and the model performances were compared. As a result, the relationship between manufacturing parameters and mechanical properties in PC-ABS composite was experimentally revealed, and an approach for determining optimum parameters was presented. Although the mechanical properties of polymer composites produced by FDM have been investigated in the literature, studies on the comparative modeling of the integrated effects of printing speed, infill density, and raster angle on multiple mechanical properties in PC-ABS materials using machine learning have been limited. This study brings a novel approach to the literature by integrating different machine learning algorithms with FDM on PC-ABS material samples, contributing to both the optimization and reliable prediction of their mechanical properties.

## 2. Materials and Methods

In this study PC/ABS, based filament material was utilized for production through the FDM additive manufacturing technique. The specimens for tensile, bending, and impact tests were made with a Bambu Lab X1 Carbon model FDM 3D printer (Bambu Lab, Shenzhen, China). Mechanical properties of the specimens were estimated by carrying out tensile and bending tests on a Shimadzu AGS, X 10 kN universal testing machine (Shimadzu Corporation, Kyoto, Japan), whereas impact tests were done on an Instron Ceast 9350 high-energy impact testing machine (Instron, Norwood, MA, USA). Tensile strength, flexural strength, and impact energy parameters were measured during experiments. The experimental results obtained were assessed using statistical evaluation and predictive modeling methods. In this regard, both the way ANOVA as well as machine learning techniques were used to carry out thorough statistical and model-based analyses.

### 2.1. Material

Within the scope of the study, the materials section includes the FDM 3D printer (Bambu Lab, Shenzhen, China). for manufacturing the specimen, mechanical testing, dataset, machine learning models, performance metrics, and Analysis of Variance (ANOVA).

#### 2.1.1. FDM 3D Printer

**Figure 1:**
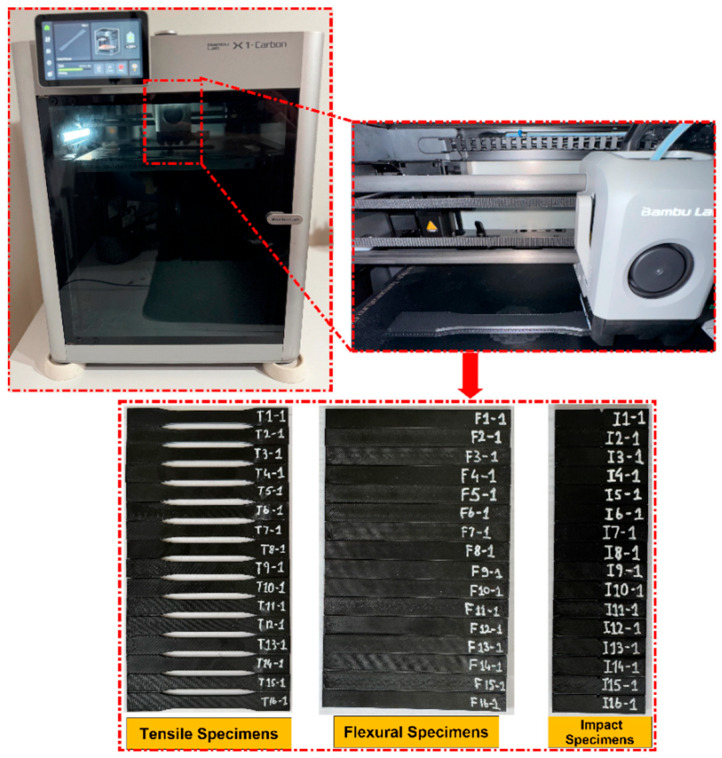
Production process of PC/ABS samples.

In this study, tensile, bending, and impact test specimens made from PC/ABS material were fabricated using the Bambu Lab X1 Carbon model FDM 3D printer (Bambu Lab, Shenzhen, China) shown in Figure 1. The FDM 3D printer has a print volume of 256 × 256 × 256 mm^3^, a hotend that can reach up to 300 °C, and a bed system that can be heated up to 120 °C. Equipped with a standard 0.4 mm diameter nozzle, the printer also supports different nozzle diameter options. It has a maximum speed of 500 mm/s and a flow capacity of up to 32 mm^3^/s. Working with 1.75 mm diameter filaments, the device is compatible with common engineering-grade thermoplastic filaments as well as fiber-reinforced composite materials. In this study, PC/ABS polymer thermoplastic was preferred because it offers high impact resistance, thermal stability, shape stability, and machinability all together. PC/ABS filament is easily machinable thanks to its high temperature resistance and good fluidity properties; it has technical specifications such as a thermal bending temperature of 120 °C, a density of 1.13 g/cm^3^, and a melt flow index of 12 g/10 min.

#### 2.1.2. Mechanical Tests

Tensile, bending, and impact tests were performed to analyze the elastic and plastic behavior of the materials under static load in detail. In this context, tensile and bending tests were carried out using a Shimadzu AGS-X 10kN machine (Shimadzu Corporation, Kyoto, Japan), while impact tests were performed using an Instron Ceast 9350 high-energy impact testing machine (Instron, Norwood, MA, USA). The clamping and holding fixtures for tensile and bending tests with a maximum load of 10 kN contain features adaptable to various sample sizes and loading conditions. The impact testing machine has an adjustable impact energy capacity in the range of 0.59–1800 J and the ability to perform tests in the speed range of 0.77–24.0 m/s and can simulate various scenarios with different hammer masses and impact heights.

#### 2.1.3. Datasets

In the dataset, samples were produced under 16 different experimental conditions for each mechanical experiment. Three repetitions were performed for each experimental condition, resulting in a total of 48 samples for each test method. Each of these samples was independently printed and tested separately. Therefore, the 48 data obtained were not derived from repeated measurements on the same sample, but from different and independent samples. This approach ensures the statistical independence of experimental data. These repetitions were performed to reduce the effect of random variability in the production process and to increase the reliability of the experimental results. The dataset consists of raster angle, infill density, and printing speed as parameters, and tensile strength, flexural strength, and impact energy as output parameters. The data obtained from tensile, flexural, and impact tests were processed using the Python 3.12.7 programming language. Pandas and NumPy libraries were used for data management and numerical processing. Pandas allows for the organization of data in a DataFrame structure and the performance of filtering, merging, and transformation operations on the data, while NumPy offers high-performance calculations with vectorized mathematical operations on multi-dimensional arrays. The scikit-learn library was used for machine learning applications. This library provides a standard structure for model creation, training, and evaluation processes, and was effectively used in data splitting, performance metric calculation, and cross-validation. Hyperparameter optimization was performed using the Optuna library. Optuna manages the hyperparameter search process with a Bayesian optimization approach and evaluates each trial according to model performance, directing it towards more suitable parameter regions. Dimensional measurements of the samples were verified using a digital caliper. It was determined that the measurements were consistent with the target design values within acceptable tolerances. In addition, experimental data were recorded directly through the data acquisition systems of the test devices, thus minimizing errors that could arise from manual data entry. In this context, measurement uncertainties were minimized as much as possible, no missing data were found in the data collection process, and the integrity of the dataset was preserved.

In this study, a data enhancement approach was applied to improve the generalization capability of machine learning models. First, the dataset was divided into 70% training and 30% test. Data enhancement was performed only on the training dataset, while the test dataset was entirely composed of real data to ensure an unbiased evaluation of model performance. Synthetic data generation was carried out using a K-Nearest Neighbor (KNN)-based interpolation method. In this context, a KNN model containing a maximum of 10 neighbors was created using the continuous variables in the training dataset. For each new data point, a random sample was selected from the training dataset, and the nearest neighbors of that sample were determined. Linear interpolation was applied between two points, with the interpolation coefficient randomly determined using the Beta distribution (β = 0.4). Furthermore, multiple interpolation was performed by selecting between 3 and 6 neighbors with a certain probability and using weights based on the Dirichlet distribution (α = 0.4). This approach allowed for the preservation of the local structure in the data space. All generated synthetic samples were constrained between the minimum and maximum values of the input parameters in the original dataset. With this method, the training dataset was expanded to 1000 samples, aiming to produce more stable and reliable results for machine learning models. In the dataset, printing speed refers to the movement speed of the print head during the production of the samples and directly affects the cooling behavior of the material and the quality of interlayer bonding. High printing speeds can cause the material to solidify before sufficient bonding occurs between the layers, leading to a weakening of mechanical properties. Raster angle defines the orientation of the infill structure relative to the build plate surface and significantly affects the distribution of stress within the printed structure. Changes in raster angle depending on the loading direction can alter the load transfer mechanism, causing differences, especially in tensile and bending performance. Infill density refers to the amount of material within the printing volume and is directly related to the internal density of the sample. Increasing the infill density increases mechanical strength due to the reduction in internal voids, while low infill density creates lighter but mechanically weaker structures. Table 2 presents the statistical properties of both input and output variables. Input parameters consist of printing speed, raster angle, and infill density, while response variables consist of tensile strength, flexural strength, and impact energy. For each variable, the sample size, mean, standard deviation, minimum, and maximum values are given.

**Figure 2:**
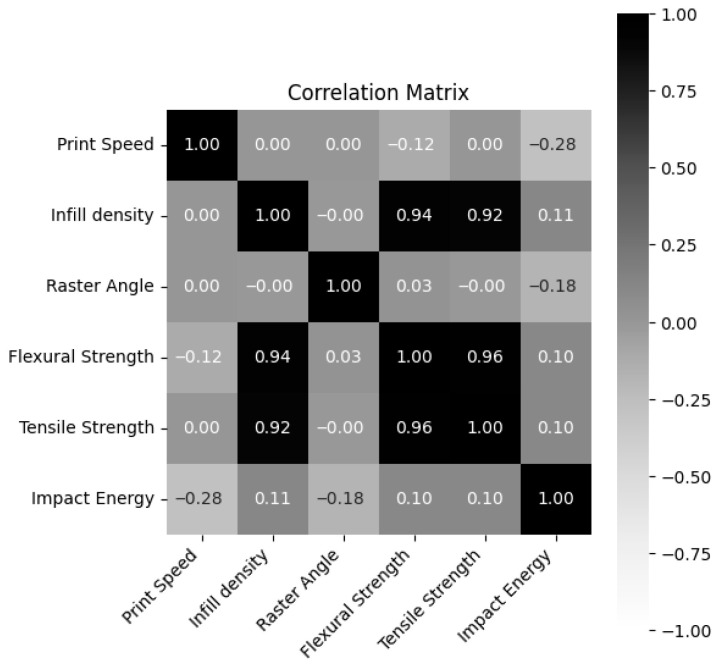
Correlation matrix of the dataset.

Figure 2 presents the correlation matrix of the dataset obtained from mechanical experiments. The relationships between variables were quantitatively evaluated using Pearson correlation coefficients (r) and their corresponding *p*-values. The significance level was set at α = 0.05, and relationships with *p* < 0.05 were considered statistically significant. According to the results, there are strong and statistically significant positive correlations between infill density and flexural strength (r = 0.94, *p* < 0.001) and tensile strength (r = 0.92, *p* < 0.001). In addition, a very high level of positive correlation was observed between flexural strength and tensile strength (r = 0.96, *p* < 0.001). In contrast, the effect of raster angle on mechanical properties was found to be more limited compared to other parameters, and no statistically significant relationship was found between this parameter and mechanical properties (*p* > 0.05). A negative relationship was observed between printing speed and impact energy (r = −0.28, *p* = 0.053), but this relationship was determined to be statistically insignificant. However, this suggests that there may be a tendency for impact resistance to decrease with increasing printing speed.

#### 2.1.4. Machine Learning Algorithms

In this study, data-driven machine learning approaches were used to model and predict the nonlinear relationships between production parameters and mechanical properties in PC-ABS samples produced by FDM [40]. In this study, Decision Tree, Random Forest, K-Nearest Neighbors, and Multilayer Perceptron algorithms were chosen because of their ability to model nonlinear relationships on limited experimental datasets, their capacity to compare the generalization performance of different learning approaches (tree-based, distance-based, and artificial neural network-based), and their successful results in the literature. Furthermore, these models are widely used in the academic literature due to their potential to predict complex interactions between manufacturing parameters and mechanical properties with high accuracy. These algorithms have been widely used to predict mechanical properties such as tensile strength, flexural strength, and impact strength in FDM processes due to their capability to capture complex nonlinear relationships and parameter interactions [41]. Furthermore, these methods demonstrate robustness against multicollinearity problems, increasing model stability, reducing variance by combining Decision Trees, and minimizing the risk of overfitting in high-dimensional parameter spaces. In this study, different machine learning algorithms were used to predict the mechanical properties of PC-ABS samples produced by FDM. Specifically, Decision Tree was chosen for its interpretability, Random Forest for its high accuracy and robustness against overfitting, KNN for its ability to capture local data patterns, and MLP for its capacity to model complex nonlinear relationships. Table 3 provides detailed information on the description, theoretical basis, and mathematical formulation of the Decision Tree, Random Forest, K-Nearest Neighbors, and Multilayer Perceptron Regressor machine learning algorithms used.

#### 2.1.5. Performance Evaluation Metrics

In this study, the prediction performance of the Decision Tree, Random Forest, K-Nearest Neighbors, and Multilayer Perceptron Regressor machine learning algorithms used in the training process was evaluated using Mean Absolute Error (MAE), Mean Absolute Percentage Error (MAPE), Mean Squared Error (MSE), Root Mean Squared Error (RMSE), and R-squared (R^2^) performance evaluation metrics.

MAE calculates the average of the absolute differences between the actual values and the values predicted by the model, and a lower MAE value indicates higher prediction accuracy [47]. MAPE expresses the average percentage error of the predictions relative to the true values, thereby providing an easily interpretable measure of model accuracy in percentage terms [48]. MSE calculates the average of the squared differences between the actual and predicted values and is particularly sensitive to large errors, making it useful for assessing the overall magnitude of prediction error. RMSE, obtained by taking the square root of the MSE value, expresses the prediction error in the same unit as the dependent variable, which facilitates interpretation and comparison [49]. Finally, R^2^ indicates the extent to which the model explains the total variance in the dependent variable; values closer to 1 indicate a better fit and stronger explanatory power [48]. The mathematical expressions of these performance evaluation metrics are given in Equations (1)–(5) [47,48,49,50]. In these equations, *n* denotes the number of samples, y_i_ represents the actual values, and ${\hat{y}}_{i}$ represents the predicted values.

(1) $$MAE=\frac{1}{n}\sum \left|y_{i}-{\hat{y}}_{i}\right|$$

(2) $$MAPE=\frac{100}{n}\sum \left|\frac{y_{i}-{\hat{y}}_{i}}{y_{i}}\right|$$

(3) $$MSE=\frac{1}{n}\sum {\left(y_{i}-{\hat{y}}_{i}\right)}^{2}$$

(4) $$RMSE=\sqrt{\frac{1}{n}\sum {\left(y_{i}-{\hat{y}}_{i}\right)}^{2}}$$

(5) $$R^{2}=1-\frac{\sum {\left(y_{i}-{\hat{y}}_{i}\right)}^{2}}{\sum {\left(y_{i}-\bar{y}\right)}^{2}}$$

Each of the performance evaluation metrics used in the study contributes to identifying the strengths and weaknesses of the models by detecting different types of errors. Therefore, a comprehensive analysis was conducted by considering all metrics together to examine model performance holistically and in depth.

#### 2.1.6. Statistical Analysis (ANOVA)

The one-way Analysis of Variance (ANOVA) used in this study is a fundamental analytical method that evaluates whether different independent variables and their potential effects create statistically significant differences on a single dependent variable. This method is used as an important analytical tool, especially in revealing statistically significant and strong effects of independent variables and their combinations of interactions on the dependent variable [51]. In this study, ANOVA was used to test whether there was a statistically significant difference between the means of the independent variable levels on a single dependent variable. Tensile, flexural strength, and impact energy measurements were evaluated by comparing the mean values for each parameter level; the total variance was separated into within-group and between-group components and tested using the F-statistics. The statistical significance level was set at *p* < 0.05, and a 95% confidence interval was reported. According to the ANOVA results, the parameters with the highest impact on mechanical properties were ranked, and their effect sizes were calculated to strengthen inferences regarding the practical importance of the findings. This approach allowed for the systematic presentation of relationships between variables and the clear identification of which factors have the greatest impact on mechanical performance; thus, it was aimed that the obtained data would contribute both to updating the theoretical framework and improving applications.

### 2.2. Method

This study methodology outlines the production of PC-ABS specimens by the FDM additive manufacturing method, the performance of mechanical experiments to determine the tensile, flexural, and impact properties, the drafting of machine learning models for the prediction of manufacturing parameters and mechanical performance, and finally the use of statistical analysis techniques to examine the correlations between process parameters and mechanical properties.

#### 2.2.1. Design and Fabrication of Test Specimens

In this study, tensile, bending, and impact test specimens were designed according to ASTM D638-14 [52], ASTM D790-17 [53], and ASTM D6110-18 [54] standards, respectively. Modeling was completed adhering to standard dimensions, the specimens were transferred to Bambu Studio 1.8.1 software on a Bambu Lab X1 Carbon printer, and the slicing process was performed according to the determined FDM printing parameters. The main FDM parameters examined in manufacturing were printing speed, infill density, and raster angle; all level combinations and the specimen coding scheme are given in Table 4. To ensure the repeatability of the experimental setup, some printing parameters were kept constant throughout the experiments. The nozzle diameter was set at 0.40 mm and the layer height at 0.20 mm. The nozzle temperature was kept constant at 290 °C and the print bed temperature was kept at 90 °C. The number of walls was set at 2, the number of top layers at 3, and the number of bottom layers at 3. All other printing parameters, including fan settings and reservoir conditions, were kept constant throughout the production process. After printing, support structures were removed in a controlled manner, edge burrs were removed, and out-of-tolerance/damaged specimens were eliminated. The material used was PC/ABS.

#### 2.2.2. Mechanical Experiments

**Figure 3:**
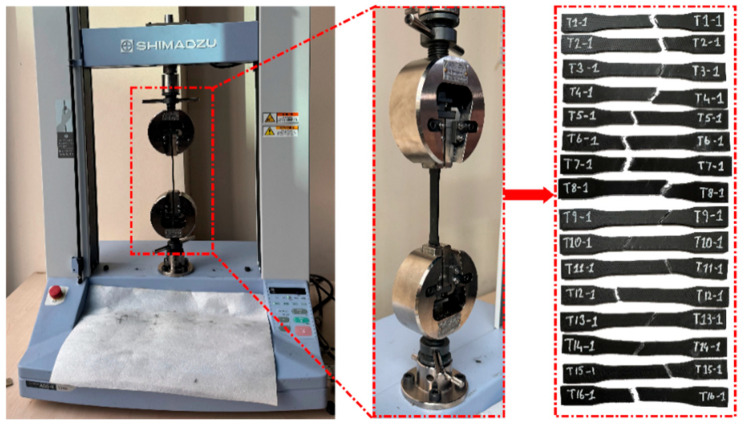
Tensile testing process.

Tensile tests were performed according to ASTM D638-14 standard [52] on a 10 kN capacity Shimadzu AGS-X universal testing machine (Shimadzu Corporation, Kyoto, Japan), as shown in Figure 3. The tests were conducted under room conditions. Specimens were placed in the self-aligning jaws of the machine, and alignment errors were minimized by applying a low preload before each test. The test speed was kept constant at 1 mm/min, and force–strain data were recorded in real time by the machine’s computer-based data acquisition system. The initial dimensions of the specimens were 57 mm in length, 13 mm in width, and 3.2 mm in thickness. These dimensions were verified at three different points on each specimen using a digital caliper before testing. The modulus of elasticity was obtained from the stress–strain slope in the elastic region. Linear elastic behavior was assumed in all calculations.

**Figure 4:**
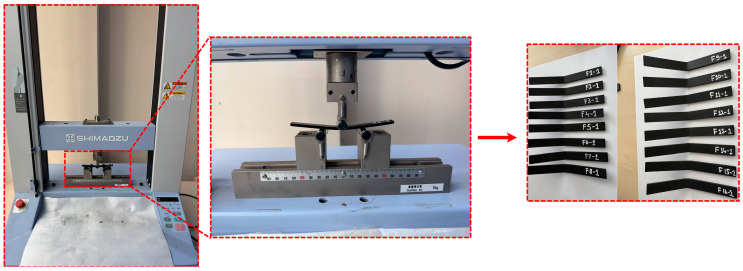
Bending test process.

Bending tests were performed in accordance with the ASTM D790 standard, using a 10 kN capacity Shimadzu AGS-X universal testing machine under room conditions, as shown in Figure 4. Prior to testing, thickness d = 3.20 mm and width b = 12.70 mm was measured on at least three points on each specimen and averaged. Testing was conducted in a three-point bending configuration with a support span L = 52 mm and a loading rate of 1 mm/min. This ensured a span/thickness ratio L/d ≈ 16.25, meeting the ≥16:1 criterion specified in ASTM. Support and load bushings were used with radii conforming to the standard, and low preload was applied for alignment. *n* = 3 repetitions were performed for each parameter group, and force–deflection data were recorded simultaneously with the machine’s data acquisition system.

**Figure 5:**
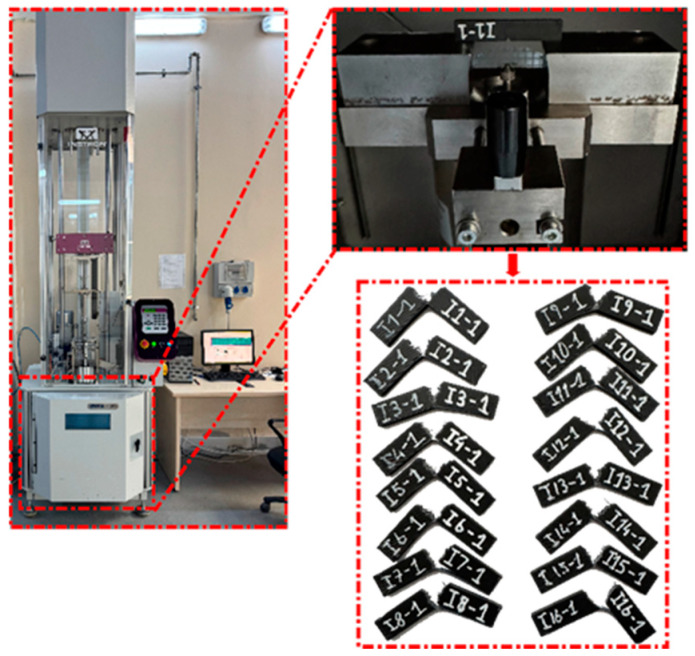
Impact testing process.

Impact strength tests were performed in accordance with the ASTM D6110-18 standard [54] using an Instron Ceast 9350 impact testing machine (Instron, Norwood, MA, USA) under room conditions, as shown in Figure 5. The machine was configured via its software to have an initial impact velocity of v = 2 mm/s. During the experiment, impact load, displacement, and energy absorption were simultaneously recorded using the machine’s computer-based data acquisition system. At least *n* = 3 repetitions were performed for each parameter group. The cross-sectional dimensions of the specimens were measured on at least three points on each specimen before the experiment and averaged; thickness d = 3.20 mm and width b = 12.70 mm were determined. For unnotched specimens, this was taken as the cross-sectional area in the impact zone. Impact strength was calculated using the obtained fracture energy data. The calculations followed the data processing steps and the assumption of small strain as stipulated by ASTM D6110.

#### 2.2.3. Machine Learning Models

This study applies machine learning algorithms to predict the mechanical properties, such as tensile and flexural strength, of PC/ABS samples produced by the FDM method. Four different machine learning algorithms were used for training: Decision Tree, Random Forest, K-Nearest Neighbors, and Multilayer Perceptron Regressor. Printing speed, infill density, and raster angle were used as input parameters for the machine learning algorithms. Furthermore, the dataset was split into 80% training/20% test ratios to test model generalizability, and all analyses were performed according to this split. To improve model performance, hyperparameter optimization was performed using the Optuna search module, and early stopping was enabled with MedianPruner. The objective function was defined to maximize the mean R^2^ value of GroupKFold (k = 5) only on the training set; the test set was not included in the optimization. Search spaces were determined based on commonly used limits in the academic literature. The hyperparameter and range values used in the study are given in Table 5. Furthermore, the multicollinearity between the input variables was quantitatively evaluated using Variance Inflation Factor (VIF) analysis. According to the results, the VIF value was calculated as 17.90 for printing speed, 9.28 for infill density, and 16.66 for raster angle. These values indicate a high level of linear correlation between some variables. However, since the machine learning algorithms used in this study are not based on linear parameter estimation, they are less susceptible to multicollinearity problems and provide reliable model performance despite the correlation between the input variables.

#### 2.2.4. Statistical Analysis Methods

In this study, the effect of FDM processing parameters on the mechanical properties of PC/ABS filament material, such as tensile, bending, and impact strength, was statistically evaluated using one-way Analysis of Variance (ANOVA). ANOVA determines which processing parameters have a statistically significant effect on the outputs by comparing the observed variation between different experimental groups with the within-group variation. For this purpose, samples were produced at different parameter levels, and the obtained experimental data were analyzed. Results below the *p* < 0.05 threshold showed that the relevant processing parameter had a significant effect on the mechanical properties; the total variance was decomposed into between-group and within-group components; and the significance level was calculated using the F-statistics. All statistical evaluations were performed using Minitab 22.3 software. Analyses were conducted under the assumption of independent samples; randomness, experimental design, and sample sizes were ensured during the design phase. This approach reliably and comparably revealed the effect of parameter variations on the mechanical strength of PC/ABS material.

## 3. Results

### 3.1. Tensile Test

Tensile tests of PC-ABS specimens produced by the FDM method were evaluated by averaging three repetitions. As shown in Table 6, the highest tensile strength among the specimens was obtained in specimen number 9 with a value of 40.71 MPa. The highest elongation at break was observed in specimen number 2 with 11.71%. The lowest performance was observed in specimen number 4, where the tensile strength was determined as 25.10 MPa. In general, the results show that the applied parameter combinations play a decisive role in both strength and ductility.

**Figure 6:**
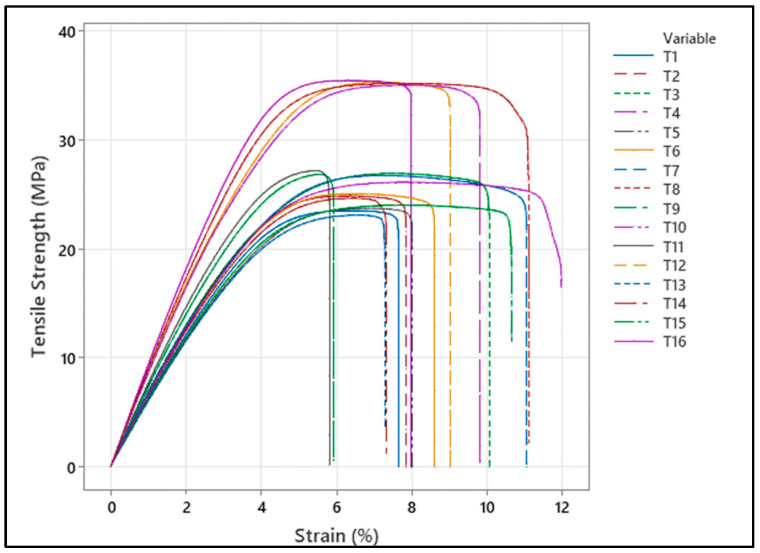
Tensile strength–strain graph.

Figure 6 presents the stress–strain curves of sample groups T1–T16 collectively. In all curves, the distinctly linear initial region indicates elastic behavior, followed by a decrease in slope, yield transition, and then reaching the maximum stress value. The stress drop before fracture shows the ductile fracture character of the PC/ABS material and the sensitivity of interlayer bonding to compression parameters. The graphs generally show a balance between strength and ductility, with high strength occurring in some samples accompanied by more limited elongation. Furthermore, the high tensile strength and acceptable elongation at fracture exhibited by samples T4, T8, T12, and T16 suggest that the relevant parameter combinations are more advantageous in terms of tensile performance.

### 3.2. Bending Test

Tensile tests of PC-ABS specimens produced by the FDM method were evaluated by averaging three repetitions. As shown in Table 7, the highest maximum stress among the specimens was obtained in specimen F8 with a value of 66.13 MPa. The highest maximum percentage elongation was observed in specimen F4 with 6.10%. The lowest maximum stress was determined in specimen F5 with 45.13 MPa, while the lowest maximum percentage elongation was observed in specimen F11 with 4.16%. Overall, the results show that the processing parameters created significant differences in the maximum stress values, while the variation in the maximum percentage elongation remained more limited.

**Figure 7:**
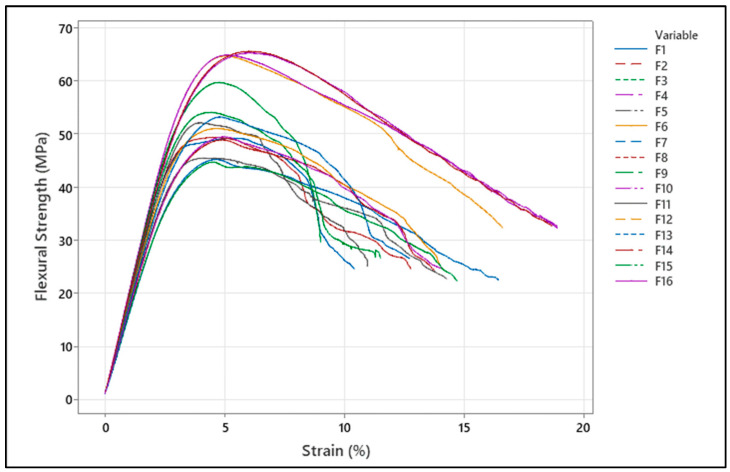
Flexural strength–strain graph.

Examination of the bending stress–percentage strain curves in Figure 7 reveals that all specimens initially exhibit a linear elastic region, then reach the yield transition, and after the maximum stress value, the stress gradually decreases. This behavior indicates that the PC/ABS material exhibits ductile fracture characteristics under three-point bending loads. As a general trend, specimens with high strength and rigidity show more limited pre-fracture strain, while specimens with lower strength exhibit increased deformation capacity. These findings demonstrate that manufacturing parameters directly affect the balance between rigidity and ductility.

### 3.3. Impact Test

Table 8 shows that the average impact energy values range from approximately 1.04 J to 2.47 J. The highest average impact energy was determined in sample group I6 as 2.467 J, followed by samples I7 at 2.249 J and samples I12 at 1.830 J. The lowest values were observed in sample group I14 at 1.042 J and sample group I13 at 1.085 J. This distribution indicates that the energy absorption capacity of PC/ABS material under impact loads is sensitive to production parameters, and variables such as infill density and printing speed directly affect impact performance.

### 3.4. Analysis of Variance (ANOVA)

#### 3.4.1. Tensile Test ANOVA Analysis

The results of the Analysis of Variance (ANOVA) are presented in Table 9. Accordingly, the main effects of printing speed, infill density, and raster angle on tensile strength, as well as the interactions between these factors, were statistically evaluated. The results show that all main factors have a significant effect on tensile strength (*p*-value < 0.05). In terms of contribution percentage, the most dominant parameter was the infill density, explaining 53.66% of the total variance. This was followed by the printing speed × infill density interaction with a contribution of 22.21% and the infill density × raster angle interaction with a contribution of 12.72%. The contribution of the main effect of printing speed was determined to be 1.67%, and the contribution of raster angle was 0.17%. However, the printing speed × raster angle interaction stands out with a contribution of 9.19%. Although the *p*-value of the triple interaction term (printing speed × infill density × raster angle) is 0.01, its contribution percentage is limited (0.17%). According to the model summary, the obtained R^2^ is 99.77%, the corrected R^2^ is 99.67%, and the estimated R^2^ is 99.49%, indicating that the established model explains the data with very high accuracy and has strong generalization performance.

**Figure 8:**
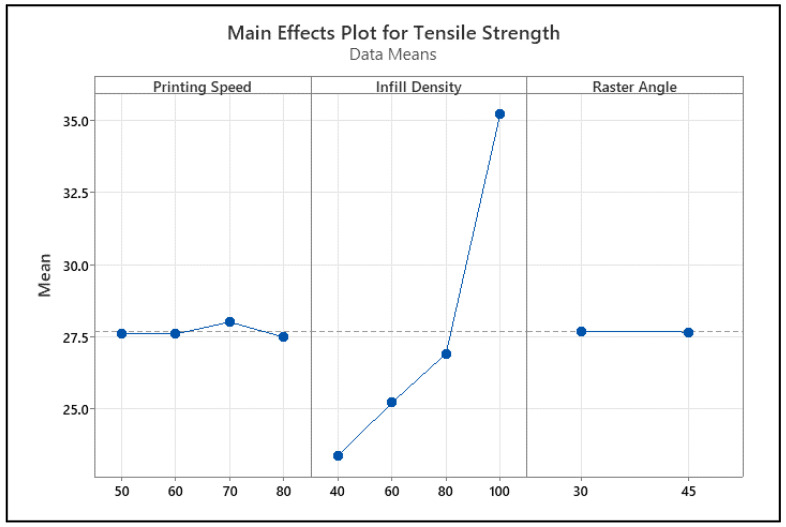
Main impact levels of tensile test. The dotted line represents the overall mean value.

The tensile strength main effect graph in Figure 8 shows that infill percentage is the most dominant factor. As the infill percentage increases from 40% to 100%, the tensile strength increases significantly, indicating that the load-carrying capacity increases with decreasing void ratio. In contrast, printing speed in the 50–80 mm/s range shows a limited effect, and raster angle (30° and 45°) does not create a significant change in tensile strength.

**Figure 9:**
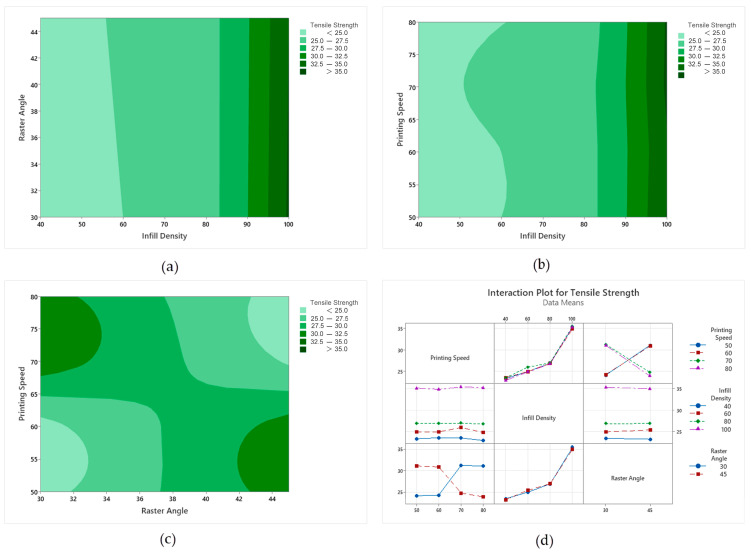
Main impact and contour graphs of tensile specimens: (**a**) raster angle–infill density, (**b**) printing speed–infill density, (**c**) printing speed–raster angle, (**d**) interaction graph of parameters.

In Figure 9a (infill density–raster angle), it is observed that the tensile strength increases significantly as the infill density increases. While the strength remains in the 25–30 MPa range at low infill density, it is seen that the strength rises above 35 MPa as the infill density increases to approximately 85–100%. In the same graph, the variation in the raster angle between 30° and 45° does not significantly change the general trend; its effect remains secondary. In Figure 9b (infill density–print speed), it is understood that the infill density is the main determinant. Although the printing speed varies between 50 and 80 mm/s, its effect on strength remains limited, and the high strength region is maintained at all speed levels, especially at high infill density. Although small fluctuations are observed depending on the printing speed at medium infill levels, this effect is weaker compared to the infill density. In Figure 9c (raster angle–print speed), the combined effect of the two parameters becomes more pronounced. While some combinations show higher strength zones, a more balanced strength band is observed at intermediate levels. This indicates that the combined effect of printing speed and raster angle can have a greater impact on the result. The interaction graphs in Figure 9d also show that infill percentage has the strongest effect on increasing tensile strength, while printing speed and raster angle contribute more limitedly. In some conditions, the change in line direction shows that interactions between factors such as printing speed and raster angle can come into play. However, it is concluded that the infill percentage is the fundamental parameter that determines the most advantageous performance window overall.

#### 3.4.2. Bending Test ANOVA Analysis

Analysis of Variance (ANOVA) results for bending strength are presented in Table 10. Accordingly, the main effects of printing speed, infill density, and raster angle, as well as the interactions between factors, were statistically evaluated. The results show that all main factors have a significant effect on bending strength (*p*-value < 0.05). In terms of contribution percentage, the most dominant parameter is infill density, explaining 43.19% of the total variance. Interaction terms also contributed significantly. The infill density × raster angle interaction contributed 18.95%, the printing speed × infill density interaction contributed 18.40%, and the printing speed × raster angle interaction contributed 15.87%. Although the contribution of the triple interaction term remained at 2.37%, it was found to be statistically significant. The main effect of printing speed was limited to 0.88%, while the main effect of raster angle was 0.06%, with a *p*-value of 0.008. According to the model summary, R^2^ values of 99.66%, adjusted R^2^ 99.51%, and estimated R^2^ 99.25% were obtained, indicating that the established model explains the data with very high accuracy and has strong generalization performance.

**Figure 10:**
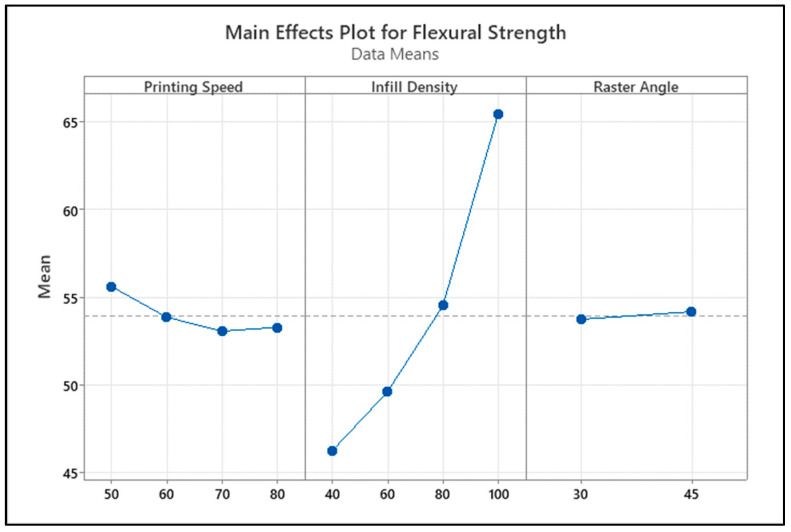
Main impact levels of bending test. The dotted line represents the overall mean value.

Examining the main effect graph on flexural strength given in Figure 10, it is clearly seen that the strongest effect on the process comes from the infill percentage. It was determined that, as the infill percentage increased from 40% to 100%, the flexural strength increased almost linearly, and the highest strength value was reached at 100% infill density. Printing speed, on the other hand, has a limited effect on flexural strength; the strength decreases slightly between 50 and 70 mm/s, but the overall trend does not show a high linear relationship. The effect of the raster angle factor is quite weak; no significant change in flexural strength is observed between 30° and 45° levels. These graph findings reveal that the primary determinant of flexural strength is the infill percentage, that the printing speed has a secondary effect, and that the effect of the raster angle remains limited.

**Figure 11:**
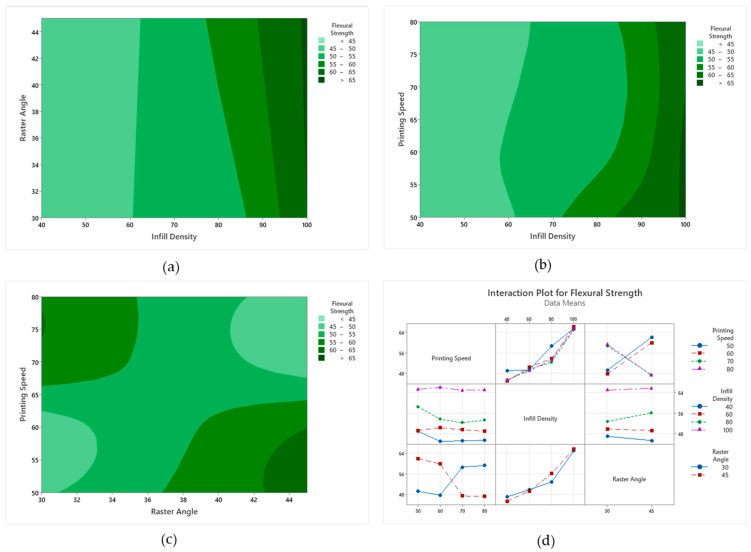
Main effect and contour graphs of the bending samples: (**a**) raster angle–infill density, (**b**) printing speed–infill density, (**c**) printing speed–raster angle, (**d**) interaction graph of parameters.

When the contour diagrams and interaction graphs of bending strength in Figure 11 are evaluated together, it is clearly seen that the strongest parameter determining bending strength is the infill density. In Figure 11a, while the bending strength generally remains in the 45–55 MPa range at an infill density of 40–60%, it is observed that the strength increases to 60–65 MPa and above as the infill density rises to 85–100%. In this graph, the variation in the raster angle between 30° and 45° shows a more limited effect compared to the infill density. Figure 11b presents the combined effect of infill density and printing speed. While higher flexural strength regions are maintained at all speed levels at high infill percentages, the effect of printing speed becomes more pronounced at medium infill levels. However, the general trend shows that the strength increases with increasing infill density. In Figure 11c, the interaction between raster angle and printing speed becomes more apparent. It has been observed that flexural strength increases with some speed and raster angle combinations, while it remains at lower levels with others. This indicates that, although raster angle and printing speed individually have a limited effect, they can significantly affect bending strength when changed together. In the interaction graphs in Figure 11d, it is seen that infill density provides the strongest increase as the main effect, and in addition, there are interaction behaviors between printing speed and raster angle. In general, it is concluded that maintaining a high infill density is critical to achieving the highest flexural strength values, while printing speed and raster angle play a secondary role as parameters supporting this performance within certain ranges.

#### 3.4.3. Impact Test ANOVA Analysis

Analysis of Variance (ANOVA) results for impact energy are presented in Table 11. Accordingly, the main effects of print speed, infill density, and raster angle, as well as the print speed × infill density interaction, were statistically evaluated. The results show that all main factors have a significant effect on energy impact. The *p*-value was obtained as 0 for print speed, 0.0006 for infill density, and 0.0097 for raster angle, all remaining below 0.05. In terms of contribution percentage, the most dominant term was the print speed × infill density interaction with 73.06%. This result reveals that impact performance is strongly dependent on the conditions created by the combination of speed and infill density, rather than individual parameters. Among the main factors, the contribution of print speed ranked second with 16.10%, while the contribution of infill density was more limited with 3.94% and raster angle with 1.31%. The error margin was determined as 5.56%. According to the model summary, R^2^ values of 94.50%, adjusted R^2^ 91.92%, and estimated R^2^ 87.62% were obtained. These values indicate that the established model explains the data with high accuracy and has acceptable generalization performance.

**Figure 12:**
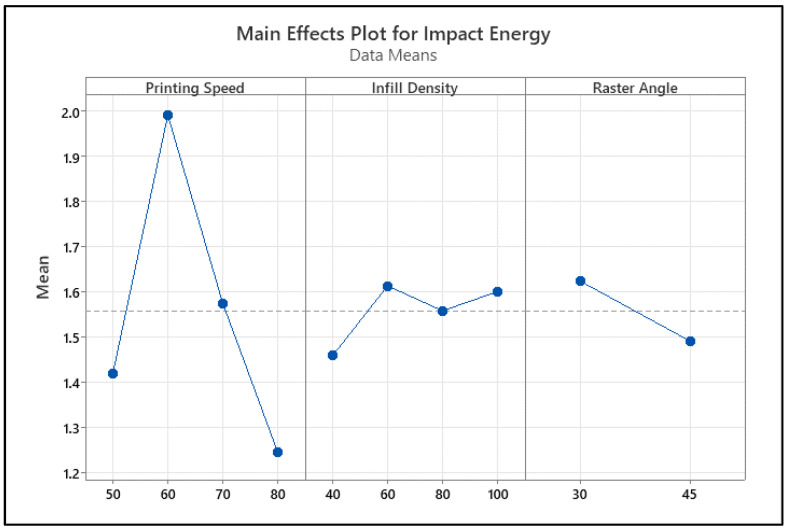
Impact test main impact levels. The dotted line represents the overall mean value.

The main effect graph for impact energy, given in Figure 12, indicates a different trend from other mechanical tests. The printing speed factor played a significant role in this test, with impact energy being low at 50 mm/s and maximum at 60 mm/s. This may be related to the more stable interlayer bonding at medium speeds. Infill percentage had a limited effect on impact energy; slight increases were observed in the 40–70% range, and a horizontal trend was observed again at 100% infill. The raster angle factor produced relatively higher impact energy at 30°, while showing a slight decrease at 45°; however, this difference indicates a weaker effect level compared to tensile and bending tests. Overall, the main effect graph shows a high sensitivity of impact energy to printing speed.

**Figure 13:**
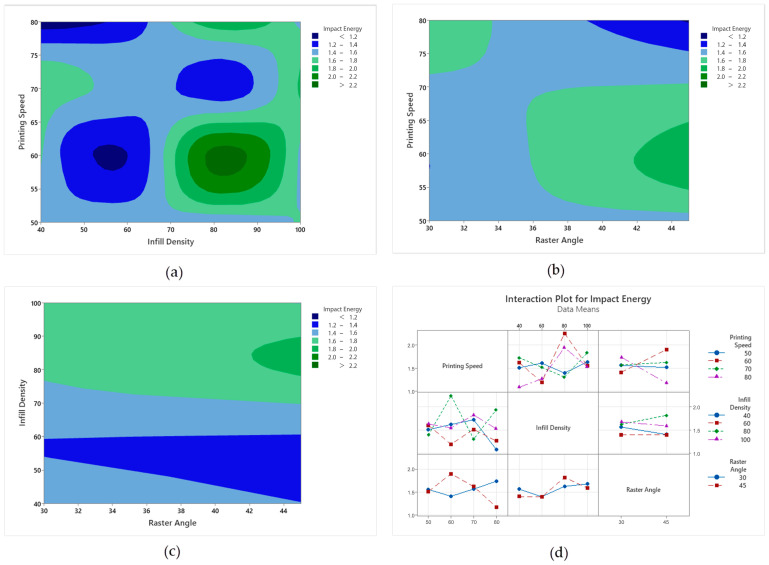
Main impact and contour graphs of impact samples: (**a**) raster angle–infill density, (**b**) printing speed–infill density, (**c**) printing speed–raster angle, (**d**) interaction graph of parameters.

When the contour diagrams and interaction graphs of impact energy in Figure 13 are evaluated together, it is seen that the impact performance changes most significantly depending on the conditions created by the combination of printing speed and infill density. In Figure 13a, it is observed that the impact of energy generally remains at lower levels at low infill density, while it can increase in appropriate speed ranges as the infill density increases. While higher impact energy regions are formed especially at medium speed levels and high infill density, areas where the energy impact decreases significantly are also seen in some speed and infill rate combinations. This indicates that the combined effect of parameters, rather than individual parameters, is decisive in impact energy. When Figure 13b,c are examined, it is understood that the effect of raster angle is more limited compared to infill density and printing speed. While a tendency to decrease in impact energy is observed at some raster angle levels as the printing speed increases, it is observed that the change in raster angle alone does not strongly change the impact energy but rather differentiates it in conjunction with other parameters. In the graph where the infill density and raster angle are evaluated together, it is noteworthy that a lower pulse energy band is formed at medium infill levels. In the interaction graphs in Figure 13d, the change in direction and convergence of the lines support the idea that there is a significant interaction between printing speed and infill density. In general, it is concluded that balancing infill rate and printing speed together is important for increasing pulse energy, while raster angle is more secondary and effective in certain combinations.

### 3.5. Findings of Machine Learning Algorithms

Table 12 presents the optimized hyperparameters and performance metrics of the MLP, Random Forest, Decision Tree, and KNN models developed for predicting tensile strength, flexural strength, and impact energy. When the results are evaluated collectively, the Decision Tree and MLP models achieved the highest prediction performance for tensile strength, exhibiting high R^2^ values together with low MAE, MSE, RMSE, and MAPE values. For flexural strength, the Decision Tree model provided the best overall performance, yielding the highest coefficient of determination and comparatively lower error metrics than the other models. In the prediction of impact energy, the MLP model produced the highest R^2^ value, while the Random Forest and Decision Tree models yielded similar error levels, indicating that impact energy was relatively more difficult to estimate accurately than the other mechanical properties. In contrast, the KNN model showed comparatively lower prediction accuracy across all target variables and demonstrated the weakest performance overall, particularly in the prediction of flexural strength and impact energy, where higher error values were observed. Overall, the findings indicate that the most suitable model varies depending on the mechanical property being predicted, and model performance should be assessed not only by the R^2^ value but also by error metrics such as MAE, RMSE, and MAPE. These results highlight the critical importance of target-specific model selection for the accurate prediction of mechanical properties in the FDM process.

**Figure 14:**
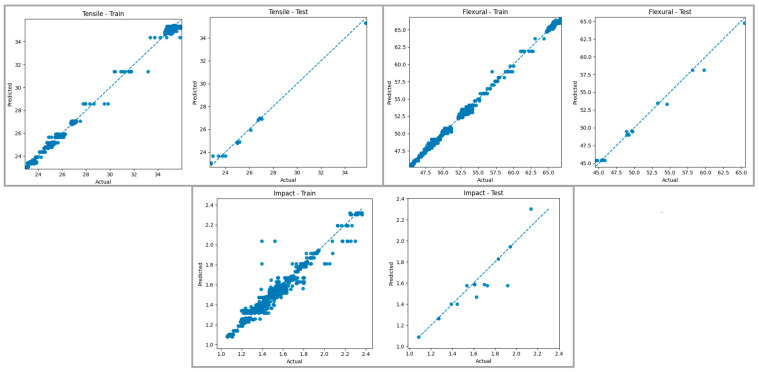
Actual–prediction graphs for the Decision Tree model. The blue dashed line represents the ideal prediction line (y = x).

Figure 14 presents a comparison of the predictions generated by the Decision Tree Regressor model for tensile strength, bending strength, and impact energy in the training and test datasets with the actual values. Examining the graphs of the training data, it is observed that the points are almost perfectly aligned with the reference line for all mechanical properties, and the model learns the training data with very high accuracy. This is due to the fact that Decision Trees divide the data space into numerous sub-regions, creating narrow decision boundaries at each leaf node, and shows a particularly pronounced tendency towards overfitting on the training data. When evaluating the graphs of the test data, it is noteworthy that, while the predictions for tensile and bending strength capture the general trend, deviations from the reference line increase in some value ranges. The more pronounced scattering observed in the test set for impact energy indicates that the model can represent the variability in this mechanical property with limited accuracy on new data.

When both graphs are evaluated together, it is seen that the Decision Tree Regressor model exhibits very high fit on the training data for tensile, bending, and impact properties, but its performance on the test data cannot be sustained at the same level. This difference between the training and test graphs reveals that the model struggles to generalize the decision limits learned during training to new data and shows a significant tendency towards overfitting. In particular, the increased scattering in the test set for impact energy indicates that the Decision Tree’s ability to generalize can weaken in complex data structures with a limited number of samples. These findings show that, although the Decision Tree Regressor method can capture the basic trend in predicting mechanical properties within the FDM process, it offers limited reliability in terms of generalization performance when used alone, and ensemble-based methods are needed for more balanced results.

**Figure 15:**
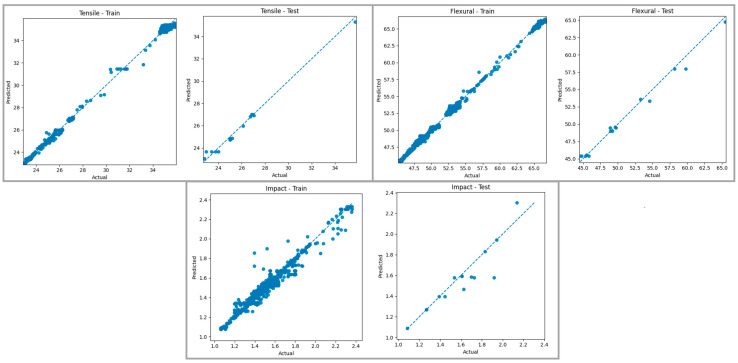
Actual–prediction graphs for the Random Tree Regressor model. The blue dashed line represents the ideal prediction line (y = x).

Figure 15 shows a comparison of the predictions produced by the Random Forest Regressor model for tensile strength, bending strength, and impact energy in the training and test datasets with the actual values. Examining the graphs of the training data, it is observed that the points are largely aligned with the reference line for all mechanical properties, and the model produces predictions with high accuracy, especially in the medium and high value ranges. This indicates that the Random Forest algorithm, thanks to its multi-tree structure, reduces the overfitting tendency seen in individual Decision Trees, thus modeling the overall trend of tensile, bending, and impact properties in a more balanced way. Evaluating the graphs of the test data, it is understood that the generalization performance of the model follows a consistent trend with the training data, and the predictions are quite close to the actual values, especially for tensile and bending strength in the medium value ranges. The limited deviation of the predictions from the actual values in some examples in the test set for impact energy is attributed to the higher variability of the data distribution for this mechanical property and the model’s ability to represent this variability with limited accuracy.

When both graphs are evaluated together, it can be said that the Random Forest Regressor model generally produces predictions with high accuracy for tensile, bending, and impact properties on both the training and test datasets, and largely successfully represents the variability structure in the dataset. In particular, the concentration of predictions around the reference line for bending strength indicates that the model has learned the relationships related to this mechanical property in a stable manner. Consistent results obtained in the medium and high value ranges for tensile strength support the model’s generalization ability, while the limited deviations observed in the test set for impact energy remain within acceptable error levels. These findings demonstrate that the Random Forest Regressor method offers a suitable, reliable, and robust modeling approach for predicting mechanical behavior within the FDM process.

**Figure 16:**
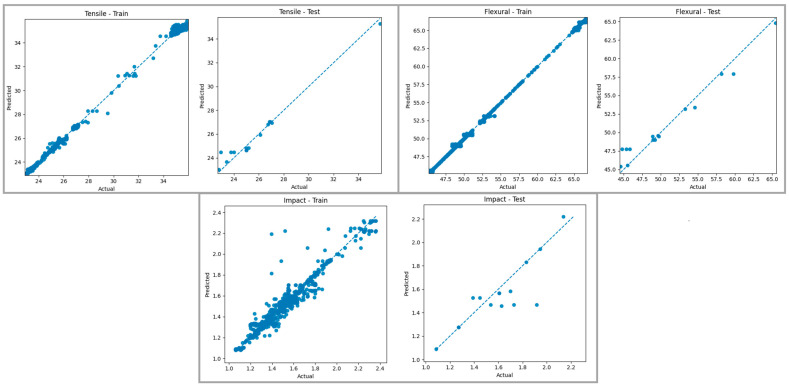
Real–prediction graphs for the KNN Regressor model. The blue dashed line represents the ideal prediction line (y = x).

Figure 16 presents a comparison of the predictions generated by the KNN Regressor model for tensile strength, bending strength, and impact energy in the training and test datasets with the actual values. Examining the graphs of the training data, it is observed that the points for tensile and bending strength are largely aligned with the reference line, and the model can produce predictions with high accuracy for these mechanical properties. It is noteworthy that the KNN algorithm achieves a successful fit, especially in the medium value ranges where data density is high, thanks to its distance-based approach between similar samples. Evaluating the graphs of the test data, it is seen that the general trend is captured for tensile and bending strength, but the predictions show limited deviations from the reference line in some value ranges. For impact energy, the scattering is more pronounced in the test set, and it is understood that the predictions are below or above the actual values in some samples. This indicates that the prediction accuracy decreases, especially in regions with increasing data sparsity, due to the KNN model’s sensitivity to the local data structure.

When both graphs are evaluated together, it can be said that the KNN Regressor model exhibits generally satisfactory prediction performance for tensile and bending strength in both the training and test datasets. The high fit obtained on the training data reveals that the model successfully learned relationships between similar samples, while the limited deviations observed in the test data indicate that its generalization ability remains at a moderate level. The increased scattering in the test set for impact energy is due to the more complex data distribution for this mechanical property and the sensitivity of the KNN approach to distant neighbors. These findings show that the KNN Regressor method offers a suitable approach for predicting tensile and bending behavior within the scope of the FDM process, but its generalization ability may be limited for more variable outputs such as impact energy.

**Figure 17:**
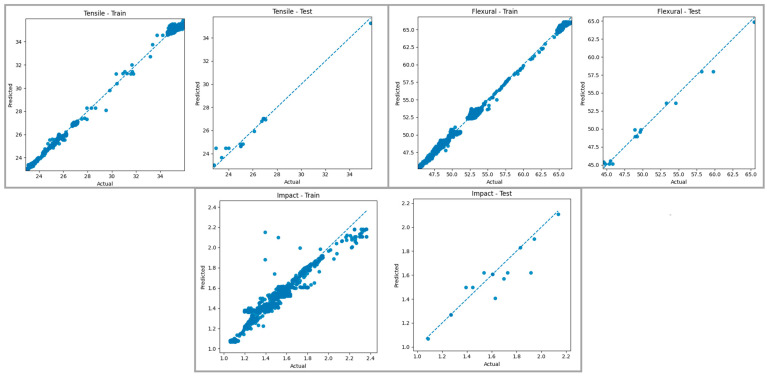
Real–prediction graphs for the MLP Regressor model. The blue dashed line represents the ideal prediction line (y = x).

Figure 17 presents a comparison of the predictions produced by the MLP Regressor model for tensile strength, bending strength, and impact energy in the training and test datasets with the actual values. When the graphs of the training data are examined, it is seen that the points are largely aligned with the reference line for all mechanical properties and that the model successfully learned nonlinear relationships. It is noteworthy that, especially for tensile and bending strength, the predictions in the medium and high value ranges are quite close to the actual values, and the scattering remains at a low level. When the graphs of the test data are evaluated, it is seen that the model maintains the general trend, but limited deviations from the reference line occur in some value ranges. The more pronounced scattering observed in the test set for impact energy is attributed to the fact that this mechanical property has a more complex data structure and the neural network model may have difficulty in the generalization process with a limited number of samples.

When both graphs are evaluated together, it can be said that the MLP Regressor model produces highly accurate predictions for tensile and bending strength in both the training and test datasets and successfully represents the nonlinear relationships in the dataset. The agreement between the training and test graphs shows that the model can largely transfer the learned relationships to new data. In contrast, the increased scattering in the test set for impact energy reveals that the model can represent the variability of this mechanical property with limited accuracy. These findings show that the MLP Regressor method offers a powerful approach for predicting tensile and bending behavior within the FDM process, but increasing the model complexity and data volume for more variable outputs such as impact energy can improve generalization performance.

## 4. Discussion

Machine learning algorithms are a frequently used technique in Fused Deposition Modeling additive manufacturing methods for predicting mechanical properties such as tensile strength, flexural strength, and impact energy. While many studies in the academic literature have focused on the mechanical properties of materials, this study employs an innovative approach that integrates ANOVA and machine learning models to predict the performance characteristics of the models with high accuracy. Within this framework, the information of the academic studies conducted is given in Table 13.

When Table 13 is examined, this study demonstrates that machine learning algorithms used to predict the mechanical properties of PC-ABS samples produced by the FDM method yield highly accurate results. ANOVA results show that manufacturing parameters have a significant effect on mechanical properties and that mechanical performance in additive manufacturing mainly depends on structural factors such as material distribution, interlayer bonding quality, and load transfer mechanisms. As stated in the literature, manufacturing parameters directly affect the internal structural density and porosity of parts produced by the FDM method, thus determining their mechanical properties [62]. However, the most striking findings obtained in the study were related to the performance of machine learning models. This result shows that nonlinear and multivariate interactions between manufacturing parameters and mechanical properties can be modeled more effectively with data-driven approaches, and that such methods provide significant advantages in the prediction and optimization of additive manufacturing processes [63]. Among the machine learning algorithms used in this study, the Random Forest model demonstrated higher accuracy in predicting tensile, bending, and impact properties, which stems from its ensemble learning structure. As stated in the literature, Random Forest improves generalization performance and reduces the risk of overlearning by reducing model variance through bootstrap sampling and random feature selection. Furthermore, its ability to combine the outputs of numerous Decision Trees allows for more effective modeling of the nonlinear and complex relationships between FDM production parameters and mechanical properties. Therefore, the Random Forest model is considered to provide more reliable prediction results, especially in limited experimental datasets [64,65].

## 5. Conclusions

This study investigates the relationships between the mechanical properties and manufacturing parameters of PC-ABS specimens produced using the Fused Deposition Modeling (FDM) additive manufacturing method, using experimental and data-driven methods. Specimens were produced using parameters such as printing speed (50–80 mm/s), infill density (40–100%), and raster angle (30–45°), and tensile, bending, and impact tests were performed. The experimental data obtained were evaluated using statistical analysis and machine learning methods.

- According to experimental results, the highest tensile strength was obtained as approximately 40.71 MPa, flexural strength as 66.13 MPa, and maximum impact energy as 2.47 J. The results showed that production parameters are decisive in mechanical performance. Increasing the infill density significantly increased the load-carrying capacity of the samples and resulted in higher strength values.
- According to the ANOVA analysis, the most influential parameter on tensile strength was the infill density with a 53.66% additive ratio. Similarly, in flexural strength, the infill density was determined to be the most dominant parameter with a 43.19% additive ratio. In impact energy, the interaction between compression speed and infill density emerged as the most significant factor with 73.06%. These results show that in the FDM production process, parameters affect mechanical performance not individually, but interactively.
- Machine learning analyses have shown that the relationship between production parameters and mechanical properties can be modeled with high accuracy. In tensile strength prediction, the Decision Tree and Random Forest algorithms showed the highest performance (R^2^ ≈ 0.99). For flexural strength, the best result was obtained with the Random Forest model (R^2^ ≈ 0.98), while Decision Tree offered similar accuracy. For impact energy, the highest explanatory power was achieved with the MLP model (R^2^ ≈ 0.83). While Random Forest performed well with balanced and low error rates, the KNN model exhibited lower accuracy compared to other methods.

In general, Random Forest can be considered the most suitable and reliable algorithm. The main reason for this is not so much that it yields the highest R^2^ value for a single target variable, but rather that it produces consistent, low-error, and balanced results across all tensile, bending, and impact properties. Furthermore, thanks to its multiple Decision Tree structure, the Random Forest algorithm reduced the overfitting tendency seen in single Decision Trees and modeled nonlinear and complex relationships in the dataset more successfully. In conclusion, it is recommended that production parameters, especially infill density, be carefully selected and that a Random Forest-based prediction approach be used in process optimization to improve the mechanical performance of PC-ABS parts. The findings obtained in this study are limited to only the parameters of printing speed, infill rate, and raster angle. Assuming other important manufacturing parameters such as nozzle temperature, layer thickness, and printing direction as constant is one of the main limitations of the study. Future studies aim to develop the machine learning-based approach proposed in this research with different algorithms, support it with larger datasets based on real experimental data, and integrate it into real-time production processes. Furthermore, it is planned to include additional parameters such as fiber orientation, fiber–matrix interaction, and interface properties in the model, especially in more complex material systems such as carbon fiber and glass fiber-reinforced polymer composites. Accordingly, by adopting a more comprehensive approach that includes not only production parameters but also material-specific structural and micromechanical variables, the goal is to increase the generalizability of the model and optimize additive manufacturing processes in a wider range of application areas.

## Figures and Tables

**Table 1:** Summary of previous machine learning studies on mechanical property prediction of FDM-produced polymer ABS materials.

Mechanical Properties	ML Technique	Ref.
Tensile, flexural and impact	Decision Tree	[37]
Tensile, flexural and surface roughness	Multiple ML models	[36]
Hardness	LR, DT, RF, AdaBoost	[38]
Tensile	Random Forest	[24]
Tensile	Decision Tree	[39]

**Table 2:** Statistical report of the dataset.

Variable	Type	Number of Samples	Mean	Standard Deviation	Minimum	Maximum
Printing Speed	input	1000	65.00	11.29	50.00	80.00
Infill Density	input	1000	70.00	22.59	40.00	100.00
Raster Angle	input	1000	37.50	7.57	30.00	45.00
Flexural Strength	output	1000	13.36	1.85	11.04	16.52
Tensile Strength	output	1000	29.51	4.90	24.31	38.26
Impact Energy	output	1000	2.00	1.00	0.05	4.39

**Table 3:** Overview of ML algorithms.

Model Name	Description	Theoretical Basis	Mathematical Formulation	Sources
Decision Trees	Decision Trees are a classification and regression method that allows the prediction of a target variable by hierarchically branching a dataset according to specific decision rules. This method offers significant advantages in terms of both explainability and interpretability due to its structurally intuitive nature and the visual interpretation of decision processes.	It is a hierarchical tree structure that performs prediction by dividing data according to its characteristics.	$H\left(S\right)=-\sum_{k=1}^{K}p_{k}{log}_{2}^{p_{k}}$ H(S): The entropy of the dataset S, representing a measure of uncertainty. S: The current dataset (i.e., the samples contained within the node). K: The total number of classes. p_k_: The probability of instances in set S belonging to the kth class. k: The class index (1,2,…,K). $G\left(S,A\right)=H\left(S\right)-\sum_{v\in \mathrm{Values}\left(A\right)}\frac{\left|S_{v}\right|}{\left|S\right|}H\left(S_{v}\right)$ G(S,A): The information gain with respect to attribute A. A: The attribute used for splitting the dataset. Values(A): The set of all possible values that attribute A can assume. |S_v_|/|S|: The weighting factor. H(S_v_): The entropy of the subset.	[38] (Veeman et al., 2023) [42] (Loh, 2011)
Random Forest	This is an ensemble learning method that enhances regression performance and strengthens model generalizability by combining the outputs of multiple Decision Trees. This approach is a machine learning algorithm that provides more effective solutions for missing data and data imbalances, as well as reducing overfitting.	It is an ensemble-based learning method for multiple Decision Trees, relying on random feature selection and bootstrap sampling.	$$\hat{y}=\frac{1}{B}\sum_{b=1}^{B}h_{b}\left(x\right)$$ B: The total number of Decision Trees employed in the Random Forest algorithm. h_b_(x): The prediction produced by the bth Decision Tree. $\hat{y}$: The final prediction of the Random Forest model.	[43,44]
K-Nearest Neighbors	The K-Nearest Neighbors algorithm is a supervised learning method that makes predictions based on similarity measures according to the positions of samples in a feature space.	Euclidean is a method that performs estimation using similarity measurement based on distance.	$$\hat{y}\left(x\right)=\frac{1}{k}\sum_{i\in N_{k}\left(x\right)}y_{i}$$ k: The number of Nearest Neighbors. N_k_(x): The set of the k Nearest Neighbors to the point x. y_i_: The actual values of these neighboring samples. $\hat{y}$(x): The predicted value for x.	[45]
Multilayer Perceptron Regressor	The Multilayer Perceptron Regressor model is a feedforward artificial neural network architecture consisting of multiple hidden layers for learning nonlinear relationships between input and output variables.	It is a feedforward network structure that learns nonlinear relationships through hidden layers and activation functions.	$$\hat{y}=\sum_{j=1}^{H}v_{j}\cdot \sigma \left(\sum_{i=1}^{p}w_{ji}x_{i}+b_{j}\right)+b_{out}$$ x_i_: Input variables. w_ji_: The weights connecting the input layer to the hidden layer. b_j_: The bias term of the hidden neuron. σ: Activation function. H: The number of neurons in the hidden layer. v_j_: The weights connecting the hidden layer to the output layer. b_out_: The output layer bias term. $\hat{y}:$ The output value predicted by the model.	[46]

**Table 4:** Input parameters.

Control Factors	Units	Levels			
Printing Speed	mm/sn	50	60	70	80
Infill Density	%	40	60	80	100
Raster Angle	°	30		45	

**Table 5:** Hyperparameter settings of machine learning algorithms used in the study.

Model	Hyperparameter	Range/Values	Description
Decision Tree	criterion	{‘squared_error’, ‘friedman_mse’}	Criterion for split.
	splitter	{‘best’, ‘random’}	Branching strategy.
	max_depth	[3, 50]	Maximum depth.
	min_samples_split	[2, 30]	Minimum number of instances required for branching.
	min_samples_leaf	[1, 30]	Number of mini-candle samples at a leaf node.
Random Forest	n_estimators	[100, 1000]	Number of trees.
	max_depth	[3, 50] or None	Maximum tree depth.
	min_samples_split	[2, 20]	Minimum number of instances required for branching.
	min_samples_leaf	[1, 20]	Number of mini-candle samples at a leaf node.
	max_features	{‘sqrt’, None}	Feature subset selection method.
k-Nearest Neighbors	n_neighbors	[1, 50]	Number of neighbors (odd numbers are given priority).
	weights	{‘uniform’, ‘distance’}	Weighting method.
	*p*	{1, 2, 3, 4, 5}	*p* = 1: Manhattan, *p* = 2: Oklid, *p* > 2: Minkowski distance.
	algorithm	{‘auto’, ‘ball_tree’, ‘kd_tree’, ‘brute’}	k-Nearest Neighbor search algorithm.
	leaf_size	[15, 100]	Leaf size for tree-based search.
Multilayer Perceptron	hidden_layer_sizes	(64)–(256.128)	Hidden layer structure (1–2 layer combinations were sought with Optuna).
	activation	{‘relu’, ‘tanh’, ‘logistic’}	Type of activation function.
	alpha	[10^−5^, 10^−2^]	L_2_ regulation coefficient to prevent overfitting.
	learning_rate_init	[10^−4^, 10^−2^]	Initial learning rate.
	solver	{‘adam’, ‘lbfgs’}	Optimization algorithm.
	early_stopping	True	Early stop has been activated.

**Table 6:** Tensile test results of the samples.

Samples	Printing Speed	Infill Density	Raster Angel	Tensile Strength (MPa)	Strain (%)
T1	50	40	30	23.12	7.74
T2	50	60	30	23.44	7.96
T3	50	80	45	23.50	10.60
T4	50	100	45	25.17	12.40
T5	60	40	30	24.98	8.13
T6	60	60	30	24.85	8.75
T7	60	80	45	26.86	10.08
T8	60	100	45	27.03	11.50
T9	70	40	45	26.90	9.70
T10	70	60	45	34.76	12.89
T11	70	80	30	34.99	6.01
T12	70	100	30	35.88	8.67
T13	80	40	45	23.43	7.65
T14	80	60	45	23.65	7.70
T15	80	80	30	23.70	6.07
T16	80	100	45	35.32	8.33

**Table 7:** Bending test results of the samples.

Samples	Printing Speed	Infill Density	Raster Ange	Flexural Strength (MPa)	Strain (%)
F1	50	40	30	49.06	10.50
F2	50	60	30	49.38	11.40
F3	50	80	45	58.63	9.79
F4	50	100	45	65.41	18.78
F5	60	40	30	45.13	13.36
F6	60	60	30	50.46	14.75
F7	60	80	45	53.75	13.04
F8	60	100	45	66.13	18.74
F9	70	40	45	45.33	16.02
F10	70	60	45	49.61	14.44
F11	70	80	30	52.36	10.76
F12	70	100	30	64.99	17.89
F13	80	40	45	45.51	16.37
F14	80	60	45	49.05	13.92
F15	80	80	30	53.37	11.00
F16	80	100	30	65.22	18.61

**Table 8:** Impact test results of the samples.

Samples	Printing Speed	Infill Density	Raster Angle	Impact Energy (J)
I1	50	40	30	1.517
I2	50	60	30	1.320
I3	50	80	45	1.349
I4	50	100	45	1.494
I5	60	40	30	1.698
I6	60	60	30	2.467
I7	60	80	45	2.249
I8	60	100	45	1.551
I9	70	40	45	1.537
I10	70	60	45	1.626
I11	70	80	30	1.308
I12	70	100	30	1.830
I13	80	40	45	1.085
I14	80	60	45	1.042
I15	80	80	30	1.328
I16	80	100	30	1.529

**Table 9:** Analysis of Variance (ANOVA) results of tensile specimens.

Source	DF	Contribution Percentage	Adj SS	Adj MS	F-Value	*p*-Value
Printing Speed	3	1.67	21.24	7.08	100.68	0
Infill Density	3	53.66	680.48	226.82	3224.37	0
Raster Angle	1	0.17	2.20	2.20	31.37	0
Printing Speed * Infill Density	9	22.21	281.64	31.29	444.84	0
Printing Speed * Raster Angle	3	9.19	116.61	38.87	552.58	0
Infill Density * Raster Angle	3	12.72	161.40	53.80	764.78	0
Printing Speed * Infill Density * Raster Angle	9	0.17	2.20	0.24	3.48	0.01
Error	32	0.17	2.25	0.07	0	0
Model Summary	R^2^	%99.77	R^2^ (adj)	%99.67	R^2^ (pred)	%99.49

* indicates interaction between factors.

**Table 10:** Analysis of Variance (ANOVA) results of bending samples.

Source	DF	Seq SS	Contribution Percentage	Adj SS	Adj MS	F-Value	*p*-Value
Printing Speed	3	31.36	0.88	0.89	10.45	37.79	0
Infill Density	3	1523.46	43.19	43.30	507.82	1835.48	0
Raster Angle	1	2.17	0.06	0.06	2.17	7.87	0.008
Printing Speed * Infill Density	9	648.97	18.40	18.44	72.10	260.63	0
Printing Speed * Raster Angle	3	559.83	15.87	15.91	186.61	674.48	0
Infill Density * Raster Angle	3	668.56	18.95	19.00	222.85	805.48	0
Printing Speed * Infill Density * Raster Angle	9	83.78	2.37	2.38	9.30	33.64	0
Error	32	8.85	0.25	0	0.27	0	0
Model Summary	R^2^	%99.66	R^2^ (adj)	%99.51	R^2^ (pred)	%99.25	

* indicates interaction between factors.

**Table 11:** Analysis of Variance (ANOVA) results of impact samples.

Source	DF	Seq SS	Contribution Percentage	Adj SS	Adj MS	F-Value	*p*-Value
Printing Speed	3	0.64	16.10	17.05	0.21	30.87	0
Infill Density	3	0.15	3.94	4.18	0.05	7.56	0.0006
Raster Angle	1	0.05	1.31	1.39	0.05	7.57	0.0097
Printing Speed * Infill Density	9	2.91	73.06	77.37	0.32	46.68	0
Error	32	0.22	5.56		0.0069		
Model Summary	R^2^	%94.50	R^2^ (adj)	%91.92	R^2^ (pred)	%87.62	

* indicates interaction between factors.

**Table 12:** Hyperparameter ranges, selected optimum values, and performance summaries of the machine learning models.

Model	Hyperparameters	Range	Tensile	Flexural	Impact
Decision Tree	max_depth	[3, 50]	8	11	11
	min_samples_split	[2, 30]	12	8	17
	min_samples_leaf	[1, 30]	10	1	4
Model Summary			R^2^ 0.99, MAE 0.22, MSE 0.07, RMSE 0.27, MAPE 0.89	R^2^ 0.98, MAE 0.45, MSE 0.43, RMSE 0.65, MAPE 0.86	R^2^ 0.81, MAE 0.07, MSE 0.01, RMSE 0.11, MAPE 4.05
Random Forest	n_estimators	[100, 1000]	500	760	130
	max_depth	[3, 50] or None	5	12	None
	min_samples_split	[2, 20]	2	2	8
	min_samples_leaf	[1, 20]	2	2	2
Model Summary			R^2^ 0.99, MAE 0.24, MSE 0.08, RMSE 0.29, MAPE 0.95	R^2^ 0.98, MAE 0.48, MSE 0.47, RMSE 0.68, MAPE 0.92	R^2^ 0.81, MAE 0.07, MSE 0.01, RMSE 0.11, MAPE 3.98
K-Nearest Neighbors	n_neighbors	[1, 50]	3	3	9
	weights	{‘uniform’, ‘distance’}	uniform	distance	uniform
	p	{1, 2, 3, 4, 5}	2	2	1
	algorithm	{‘auto’, ‘ball_tree’, ‘kd_tree’, ‘brute’}	auto	auto	auto
Model Summary			R^2^ 0.97, MAE 0.36, MSE 0.25, RMSE 0.50, MAPE 9.92	R^2^ 0.95, MAE 0.89, MSE 1.66, RMSE 1.28, MAPE 11.57	R^2^ 0.69, MAE 0.09, MSE 0.03, RMSE 0.19, MAPE 5.72
Multilayer Perceptron	hidden_layer_sizes	(64)–(256.128)	(80, 80)	(180, 30)	(80, 50)
	activation	{‘relu’, ‘tanh’, ‘logistic’}	relu	relu	relu
	alpha	[10^−5^, 10^−2^]	0.0000215450	0.00009759155	0.0000202741
	learning_rate_init	[10^−4^, 10^−2^]	0.0088280186	0.00056257801	0.0026010161
	solver	{‘adam’, ‘lbfgs’}	adam	adam	adam
Model Summary			R^2^ 0.98, MAE 0.23, MSE 0.09, RMSE 0.30, MAPE 0.92	R^2^ 0.98, MAE 0.50, MSE 0.47, RMSE 0.69, MAPE 0.96	R^2^ 0.83, MAE 0.07, MSE 0.01, RMSE 0.11, MAPE 4.32

**Table 13:** Comparison table of ML-based and ANOVA analyses.

Author (s)	Material/Process Working Area	Algorithms Used	Target Property (ies) Predicted Property (ies)	Results	Year
Nguyen et al. [55]	PC/ABS blends—Material Extrusion (MEX)/Fused Filament Fabrication	Taguchi Design of Experiments (L9), ANOVA	Tensile strength, Flexural strength, Hardness, Impact energy (Charpy)	Layer thickness was the most influential parameter on ultimate tensile and flexural strength. The 70–30 PC/ABS blend showed the highest impact energy (~1.90 J).	2025
Nikzad et al. [56]	PLA Fused Deposition Modeling	AdaBoost, BR CatBoost, DT EN, GPR, GBM GLM, KR, KNN Lasso, LGBM LR, PR, RF, Ridge, SGD SVR, XGBoost	Tensile strength	The CatBoost algorithm gave the best result with R^2^ = 94.46% accuracy.	2025
Mahapatra et al. [57]	KF-ABS Fused Deposition Modeling	RF, SVM, LR, NN, SGD	Tensile strength	Random Forest gave the best result (R^2^ = 0.925).	2025
Boppana and Ali [58]	Polycarbonate (PC)—Fused Deposition Modeling (FDM)	I-optimal DoE, Regression, ANN, Genetic Algorithm (GA), ANOVA	Tensile strength	Regression model R^2^ = 0.9652; ANN–GA model used for optimization.	2024
Ramiah & Pandian [37]	ABS—Fused Deposition Modeling (FDM)	Taguchi (L18), Decision Tree (ML), FAHP, COPRAS, ANOVA	Tensile strength, Flexural strength, Impact energy	Raster angle, raster width and layer thickness were the most influential parameters. Optimal results: Tensile ≈ 28.42 MPa, Flexural ≈ 99.74 MPa, Impact ≈ 0.282 J.	2023
Jatti et al. [59]	PLA—Fused Deposition Modeling (FDM)	XGBoost, Random Forest, Decision Tree, SGD	Flexural strength	XGBoost showed the best performance (R^2^ = 0.77); SGD achieved the highest F1-score (0.86).	2023
Ranjan et al. [60]	ABS Fused Filament Fabrication	RF, LR, SVM and AdaBoost Regression	Flexural Strength	Random Forest gave the best result (R^2^ = 0.999), and ANOVA showed infill pattern as the most significant factor.	2023
Kumar et al. [26]	ABS–PC polymer blend filament—FDM	GA-ANN, GA-RSM, RSM, ANOVA	Tensile strength	GA-ANN achieved high prediction accuracy (R^2^ ≈ 0.996). Maximum tensile strength ≈ 50.11 MPa.	2023
Pawar and Dolas [61]	PC-ABS—Fused Deposition Modeling (FDM)	Response Surface Methodology (RSM), Regression analysis, ANOVA	Flexural strength	Regression model achieved high accuracy (R^2^ = 96.70%). Optimal parameters (0.14 mm layer thickness, 100% infill, horizontal orientation) yielded flexural strength ≈ 48.29 MPa and surface roughness ≈ 3.58 µm.	2022
In the study	PC-ABS—Fused Deposition Modeling (FDM)	Decision Tree, Random Forest, K-Nearest Neighbors, MLP Regressor, ANOVA	Tensile strength Flexural strength Impact energy	Hyper parameters tuning Random Forest gave the best prediction tensile strength (R^2^ ≈ 0.99), Flexural strength (R^2^ ≈ 0.98), and Impact energy (R^2^ ≈ 0.81), and ANOVA identified infill density as the most significant factor.	2026

## Data Availability

The original contributions presented in this study are included in the article. Further inquiries can be directed to the corresponding author.
